# Late presenters among persons with a new HIV diagnosis in Italy, 2010–2011

**DOI:** 10.1186/1471-2458-13-281

**Published:** 2013-03-27

**Authors:** Laura Camoni, Mariangela Raimondo, Vincenza Regine, Maria Cristina Salfa, Barbara Suligoi

**Affiliations:** 1 AIDS Unit, Department of Infectious, Parasitic and Immunomediated Diseases, National Institute of Health, Viale Regina Elena 299, 00161, Rome, Italy

**Keywords:** HIV, Surveillance, Late presenters, Epidemiology, Italy

## Abstract

**Background:**

In Western Europe, about 50% of newly diagnosed HIV-positive individuals are diagnosed at a late stage disease and enter in care late (i.e. with a CD4 count ≤350 μL/μL). The aim of the present study is to analyze the characteristics and the factors associated with being diagnosed late or at an advanced stage of disease among persons with a new HIV diagnosis in Italy, in the period 2010–2011.

**Methods:**

We used individual data on new HIV diagnoses reported by the HIV surveillance system in 2010 and in 2011. Persons with CD4 ≤350 cells/μL or diagnosed with AIDS (regardless of the CD4 cell count) were defined as late presenters (LP); persons with CD4 ≤ 200 cells/μL or AIDS (regardless of the CD4 cell count) were defined as presenting with advanced HIV disease (AHD).

**Results:**

Of the 7,300 new diagnoses reported in 2010–2011 by the included regions, 55.2% were LP; among these, 37.9% was diagnosed with AIDS. Persons presenting with AHD were 37.8%.

The median age of LP was 40 years (IQR 33–48), significantly higher (p < 0.001) than that of non-LP (35 years); 73.9% were males; 30.7% were non-nationals. The median age of AHD was 42 years (IQR 35–50), 74.5% were males; 31.1% were non-nationals.

The proportion of LP among IDUs was 59.8%, among heterosexuals (HET) 61.1% and among MSM 44.3%. The proportion of AHD among IDUs was 43.6%, among HET 43.2% and among MSM 27.4%.

Factors significantly associated with being LP were: age older than 50 years (OR = 4.6 [95% CI 3.8-5.6]); having been diagnosed in Southern Italy (Southern vs Northern Italy OR = 1.5 [95% CI 1.3-1.7]) having been diagnosed in Central Italy (Central vs Northern Italy OR = 1.3 [95% CI 1.1-1.6]); being HET (HET vs MSM, OR = 1.7 [95% CI 1.5-2.0]), being non-national (Non-national vs Italian, OR 1.7 (95% CI 1.5-2.0); being IDU (IDU vs MSM, OR = 1.6 [95% CI 1.2-2.1]). The same factors were significantly associated with being AHD.

**Conclusions:**

Older people, people diagnosed in Central and Southern Italy, non nationals, and persons who acquired the infection through injecting drug use or heterosexual contact showed a higher risk of being diagnosed late. A more active offer of HIV testing and targeted interventions focussed on these populations are needed to optimize early access to care and treatment.

## Background

Effective treatment for HIV has been available in Europe since the mid 1990s and has led to a dramatic reduction in the incidence of AIDS events and HIV-related deaths. Many HIV-positive individuals are now living with HIV as a chronic condition rather than an inevitably fatal illness.

However, of the approximately 2.3 million HIV-infected individuals living in the European region, it is estimated that one in three is unaware of his/her HIV serostatus (i.e. 700,000–900,000 individuals) [1], resulting in significant levels of late diagnosis and transmission across the region. In Western Europe, 45–50% of newly diagnosed HIV-positive individuals are diagnosed and enter care late (i.e. with a CD4 count ≤350 cells/μL) [2,3].

Late presentation is associated with: increased HIV-related morbidity and mortality [4-7], shorter survival [8], poor response to treatment [9,10], increased healthcare costs [11] and increased rates of HIV transmission [12]. If a person is diagnosed early and HIV treatment is introduced early in the course of infection before severe impairment of the immune system has occurred, life-expectancy may approach that of the general population [13].

The aim of the present study is to analyze the characteristics and the factors associated with being diagnosed late or at an advanced stage of disease among persons newly diagnosed with HIV infection in Italy in 2010 and 2011.

## Methods

We used individual data on new HIV diagnoses reported in Italy in 2010–2011 to the HIV surveillance system. The HIV surveillance system is coordinated by the National AIDS Unit of the National Institute of Health (Rome) and was established in 2008 by a decree of the Ministry of Health [14] which included HIV infection to the list of mandatory notifiable infectious diseases.

Individual data on HIV diagnoses reported by clinicians are available since 2010. In 2010–2011, 19 out of 21 Italian regions provided individual information on socio-demographic characteristics, transmission mode, and CD4 count at HIV diagnosis. These regions account for 87.5% of the total Italian population. CD4 count was measured within 15 days after HIV diagnosis.

Persons with CD4 ≤350 cells/μL or diagnosed with AIDS (regardless of the CD4 cell count) were defined as late presenters (LP); persons with CD4 ≤ 200 cells/μL or AIDS (regardless of the CD4 cell count) were defined as presenting with advanced HIV disease (AHD).

The proportion of LP and AHD was calculated for every variable; the characteristics of LP and AHD were compared with those of non-LP or non-AHD and differences were analyzed with the Chi-square test. To compare median age we performed statistical analysis using Mann-Whitney *U*-test.

Variables included in the univariate analysis were: year of reporting, gender, age, nationality, area of notification, transmission mode.

In order to identify factors associated with being LP or AHD, we built two multivariate logistic regression models in which being LP or AHD was considered as dependent variable. Variables with a P value of <0.05 were entered in the model. The fitness of the final model was assessed with the likelihood ratio test. Gender was forced into the model regardless of its significance.

The statistical analysis was conducted using IBM SPSS 20.0.

## Results

### Late presenters (LP)

Of the 7,300 new HIV diagnoses reported in 2010–2011 by 19 regions, the number of CD4 cell count at HIV diagnosis was reported for 5,545 persons, of whom 3,059 (55.2%) were LP, and 2,486 non-LP. Among LP, 37.9% was diagnosed with AIDS.

Among the 3,059 LP, 73.9% were males, 30.7% non-nationals, 53.3% heterosexuals (HET), 25.7% men who have sex with men (MSM), 10.3% reported other or undetermined risk factors, and 6.4% were injecting drug users (IDU). The median age was 40 years (IQR 33–48) among LP and 35 years among non-LP (IQR 28–43) (p <0.000).

One-thousand-eight-hundred-eighty-eight (61.7%) LP were notified in Northern Italy, 474 (15.5%) in Central Italy and 697 (22.8%) in Southern Italy.

**Figure 1:**
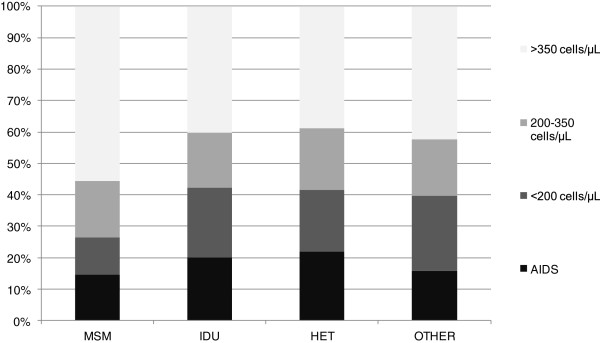
Percent distribution of CD4 counts among new HIV diagnoses by mode of transmission, Italy 2010–2011.

Among MSM, 44.3% was LP (14.5% had an AIDS diagnosis, 11.9% was diagnosed with ≤200 cells/μL and 17.9% was diagnosed with 200–350 cells/μL); among IDU 59.8% was LP (20.1% had an AIDS diagnosis, 22.3% was diagnosed with ≤200 cells/μL and 17.4% was diagnosed with 200–350 cells/μL); among HET 61.1% was LP (21.9% had an AIDS diagnosis, 19.8% was diagnosed with ≤200 cells/μL and 19.4% was diagnosed with 200–350 cells/μL) (Figure 1).

LP, compared to non-LP, underwent HIV testing because of complaining of clinical conditions associated with HIV infection (44.0% vs. 16.5%; p-value = 0.000), and pre-surgery screening (16.9% vs 14.8%; p-value = 0.037). On the opposite, having engaged in sexual risk behavior (16.6% vs. 35.0%; p-value = 0.000), being pregnant (3.3% vs 4.3%; p-value = 0.06), having a partner recently found HIV-positive (2.6% vs 4.1%; p-value = 0.002), and other parenteral blood exposures (accidental exposure to blood, syringe exchange) (7.3 vs 14.9; p-value = 0.000) were not perceived as relevant reasons for undergoing HIV testing.

Factors associated with late presentation were: increasing age, being non-national, being notified in Southern-Central Italy rather than in Northern Italy, and having been exposed to injecting drug use or heterosexual contact (Table 1).

**Table 1:** Factors associated with being a late presenter among new HIV diagnoses in Italy, 2010-2011

	**Late presenter**		**Non-late presenter**		**P**	**AOR**	**(95% IC)**
	**No.**	**% row**	**No.**	**% row**			
**Total**	3,059	55.2	2,486	44.8			
Year of reporting					n.s.		
2010	1,575	54.0	1,342	46.0			
2011	1,484	56.5	1,144	43.5			
**Gender**					n.s.		
Male	2,261	54.5	1,887	45.5		1	
Female	798	57.2	597	42.8		0.9	0.8-1.0
**Median age at diagnosis in years (IQR)**	40	(33–48)	35	(28–43)	0.000*		
**Age at diagnosis**					0.000**		
≤ 29	470	38.9	739	61.1		1	
30–34	460	50.7	448	49.3		1.7	1.4-2.1
35–39	509	54.3	429	45.7		2.1	1.7-2.5
40–49	938	61.3	593	38.7		2.9	2.5-3.4
≥ 50	678	71.1	275	28.9		4.6	3.8-5.6
**Nationality**					0.000**		
Italian	2,115	53.1	1,865	46.9		1	
Non-national	936	60.4	614	39.6		1,7	1.5-2.0
**Area of notification**					0.000**		
Northern Italy	1,888	53.2	1,658	46.8		1	
Centre Italy	474	59.1	328	40.9		1.3	1.1-1.6
**Transmission mode**					0.000**		
MSM	787	44.3	988	55.7		1	
IDU	196	59.8	132	40.2		1.6	1.2-2.1
Heterosexual	1,629	61.1	1,037	38.9		1.7	1.5-2.0
Other/not determined	447	57.6	329	42.4		1.6	1.4-2.0

* U Mann-Whitney test.

** Chi^*2*^*test.*

### Advanced HIV disease (AHD)

Among 7,300 new HIV diagnoses reported in 2010–2011, the number of CD4 cell count at HIV diagnosis was reported for 5,545 persons, of whom 2,097 (37.8%) persons presented with AHD. The proportion of males was 74.5%, 31.1% were non-nationals, 50% were HET, 23.2% were MSM, 10.8% reported other or undetermined risk factors, and 6.8% were IDU. Among AHD the median age was 42 years (IQR 34–50), whereas among non-AHD was 36 (IQR 29–43) (p =0.000).

Among IDUs the proportion of AHD was 43.6%, among heterosexuals (HET) 43.2% and among MSM 27.4%.

One-hundred-sixty-eight (61.0%) persons with AHD were reported in Northern Italy, 340 (16.2%) in Central Italy and 477 (22.8%) in Southern Italy. At multivariate analysis, the factors associated with AHD were the same as those found for LP.

Factors significantly associated with being a LP were: age older than 50 years (OR = 5.8 [95% CI 4.7-7.1]); having been diagnosed in Southern Italy (Southern vs Northern Italy OR = 1.4 [95% CI 1.2-1.6]) having been diagnosed in Central Italy (Central vs Northern Italy OR = 1.4 [95% CI 1.2-1.6]); being IDU (IDU vs MSM, OR = 1.8 [95% CI 1.4-2.3]); being HET (HET vs MSM, OR = 1.7 [95% CI 1.5-2.0]) being non-national (Non-national vs Italian, OR 1.8(95% CI 1.5-2.0).

## Discussion

Our results show that more than half of individuals with a new HIV diagnosis were LP; among these, more than two thirds were in an advanced stage of HIV disease and one third was diagnosed with AIDS.

The late HIV diagnosis and the consequent late linkage to care is a worrying scenario. Indeed, the threshold below which initiation of ART is strongly recommended by recent international and Italian guidelines is 350 CD4 cells/uL [15].

The proportion of LP at HIV diagnosis observed in our study was similar to that reported in other European countries (Denmark: 55.9%; Germany: 53%; France: 51.9%; United Kingdom: 49.2%; Spain: 45.5%) [1].

In our sample, non-nationals were more likely to be diagnosed later than Italians, as already reported elsewhere [16,17] suggesting a low access to HIV testing sites, or a poor perception of exposures at risk for HIV, or the presence of language, cultural or socioeconomic barriers. However, in Italy HIV testing, care and treatment are provided free-of-charge to both legal and illegal immigrants.

The median age was significantly higher among LP and persons with AHD compared to non-LP and non-AHD, and the probability of being LP or AHD increased with increasing age, as shown by other Italian studies [16,17]. This finding may be attributed to a lower awareness among older persons of having been exposed to HIV infection [18]. Moreover, older persons presenting with HIV-associated diseases are often misdiagnosed by general practitioners who may not consider the possibility of HIV infection in these patients.

Persons reported in Southern and Central Italy were more likely to be diagnosed late than those reported in Northern Italy, suggesting a poorer access to HIV testing centers or a lower risk perception of HIV infection. In the period 2010–2011 the incidence of new HIV diagnoses reported in Southern Italy has always been lower compared to that in Central and Northern Italy (South 3.4 per 100,000 Central 5.7 per 100,000, North 6.3 per 100,000). This finding might have led both health care personnel and general population to think that the risk of acquiring HIV infection was actually lower in Southern Italy, thus minimizing education and information campaigns aimed at HIV prevention in this area [19].

Despite the strong reduction of the HIV epidemic among IDU in Italy since 1990 [19], our results evidence a high proportion of LP and the highest proportion of persons with AHD among IDU; similar results were reported in a study conducted in Spain [20]. These findings can be explained by a lower HIV testing uptake in drug-treatment centers in the last decade [21] mainly attributable to the decline in HIV prevalence among IDU [22] and in the number of persons who inject drugs [21]. These changes may have led healthcare services to consider the HIV epidemic under control in this population thus reducing the active offer of HIV testing. This has also caused a minimization of the risk of HIV infection through unprotected sexual contact among both injecting and non-injecting drug users [23].

In our study, complaining of clinical conditions associated with HIV infection was the main reason for undergoing HIV testing among LP, confirming the relevant role of healthcare professionals (especially general practitioners) in recommending HIV testing not only in presence of AIDS-defining diseases but also for specific HIV indicator conditions [24].

Among LP, having engaged in sexual risk behavior was not a relevant reason for undergoing HIV testing, suggesting that unprotected sexual contact is not perceived as being at risk [25].

IDU and HET had a similar probability of late diagnosis, higher than that found for MSM. Also other studies conducted in different countries [18,26] report that MSM are less likely to be diagnosed late compared to IDU and HET; this finding may be associated with a higher probability among MSM of undergoing HIV testing which reflects a higher perception of sexual risk behavior [19].

Some weaknesses and strengths of this study need to be mentioned. In the first place, national data on HIV surveillance system are available only from 2010. Moreover this study shares the limitation of all surveillance systems: underreporting, missing data, timelessness and regional differences in health system.

However, our study has several advantages: it provides a picture of both LP and AHD for two recent years, is based on a robust sample of individuals, has an almost-national geographical coverage, offers information on the testing attitude of individuals exposed to HIV infection based on the reasons for HIV testing.

## Conclusions

Despite the recommendation for initiating ART early, in Italy the interventions to discover hidden HIV cases are few and the proportion of LP is quite high. In this study, more than half of new HIV diagnoses are LP, mainly persons older than 50 years of age, non-nationals, persons living in Central-Southern Italy, and persons who acquired the infection through injecting drug use or heterosexual contact. These results reinforce the importance of identifying HIV infection as early as possible and suggest the need for a more active offer of HIV testing in these groups. The presence of HIV-related symptoms represents an effective trigger for early diagnosis; general practitioners play a crucial role in the early identification of HIV-infected individuals and should consider HIV infection even among older individuals, encouraging HIV testing when appropriate. At-risk sexual behavior instead is not recognized as a sufficient reason for being tested. An effort to improve the awareness of exposures at risk is required, to optimize the early linkage to care and treatment, and to reduce HIV morbidity, mortality and transmission.

## Competing interest

The Authors declare they do not have any conflict of interest or affiliation with any organization whose financial interest may be affected by material in the manuscript, or which may potentially bias it.

## Authors’ contributions

LC has designed the study, was responsible for study coordination, data collection, statistical analysis and was the lead writer of this paper. MR helped to write the study; VR has made statistical advice; MCS has revised the paper and BS was the guarantor of the study. The regional representatives of the HIV Surveillance System were responsible for data collection. All authors have seen and approved the final version of this manuscript.

The corresponding author had full access to all the data in the study and had final responsibility for the decision to submit for publication.

## Pre-publication history

The pre-publication history for this paper can be accessed here:

http://www.biomedcentral.com/1471-2458/13/281/prepub
